# Curcuma longa L. Prevents the Loss of β-Tubulin in the Brain and Maintains Healthy Aging in Drosophila melanogaster

**DOI:** 10.1007/s12035-021-02701-6

**Published:** 2022-01-13

**Authors:** Md. Mashiar Rahman, Md. Abdullah Al Noman, Md. Walid Hossain, Rahat Alam, Selena Akter, Md. Masnoon Kabir, Mohammad Jashim Uddin, Md. Ziaul Amin, H. M. Syfuddin, Shahina Akhter, Tomasz M. Karpiński

**Affiliations:** 1 Molecular and Cellular Biology Laboratory, Department of Genetic Engineering and Biotechnology, Jashore University of Science and Technology, Jashore, 7408 Bangladesh; 2 Department of Pharmacy, Jashore University of Science and Technology, Jashore, 7408 Bangladesh; 3 Laboratory of Cancer Biology, Department of Oncology, University of Oxford, Oxford, OX3 7DQ UK; 4 Department of Biochemistry and Biotechnology, University of Science and Technology Chittagong (USTC), Foy’s Lake, Chittagong, 4202 Bangladesh; 5 Chair and Department of Medical Microbiology, Poznań University of Medical Sciences, Wieniawskiego 3, 61-712 Poznań, Poland

**Keywords:** *Curcuma longa*, β-tubulin protein, Brain, Aging, *D. melanogaster*, Longevity, Age-associated diseases, Tau protein

## Abstract

Loss of tubulin is associated with neurodegeneration and brain aging. Turmeric (*Curcuma longa* L.) has frequently been employed as a spice in curry and traditional medications in the Indian subcontinent to attain longevity and better cognitive performance. We aimed to evaluate the unelucidated mechanism of how turmeric protects the brain to be an anti-aging agent. *D. melanogaster* was cultured on a regular diet and turmeric-supplemented diet. β-tubulin level and physiological traits including survivability, locomotor activity, fertility, tolerance to oxidative stress, and eye health were analyzed. Turmeric showed a hormetic effect, and 0.5% turmeric was the optimal dose in preventing aging. β-tubulin protein level was decreased in the brain of *D*. *melanogaster* upon aging, while a 0.5% turmeric-supplemented diet predominantly prevented this aging-induced loss of β-tubulin and degeneration of physiological traits as well as improved β-tubulin synthesis in the brain of *D*. *melanogaster* early to mid-age. The higher concentration (≥ 1%) of turmeric-supplemented diet decreased the β-tubulin level and degenerated many of the physiological traits of *D*. *melanogaster*. The turmeric concentration-dependent increase and decrease of β-tubulin level were consistent with the increment and decrement data obtained from the evaluated physiological traits*.* This correlation demonstrated that turmeric targets β-tubulin and has both beneficial and detrimental effects that depend on the concentration of turmeric. The findings of this study concluded that an optimal dosage of turmeric could maintain a healthy neuron and thus healthy aging, by preventing the loss and increasing the level of β-tubulin in the brain.

**Supplementary Information:**

The online version contains supplementary material available at 10.1007/s12035-021-02701-6.

## Background

Aging is the gradual decline of organismal physiological functions caused by inevitable molecular and cellular damage. The independent or concerted action of environmental factors (diet) and genetics influence the aging process [1, 2]. This is because aging is an initial risk factor for most age-related disorders like cancer, obesity, diabetes, cardiovascular disease, arthritis, osteoporosis, and dementia [3]. Prolonging health through the slowing of aging is the most efficient strategy to prevent age-related disorders [4]. Dietary phytochemicals offer significant promise for the creation of new medication classes to treat aging-related disorders [5]. These phytochemicals can promote or demote health through the modulation of two major and evolutionary conserved nutrient-sensing IIS and TOR pathways without altering the DNA sequence [6]. Furthermore, dietary phytochemicals may activate pathways associated with aging, such as autophagy and DNA repair and counteract aging-related systemic oxidative stress and inflammation and post-translational modification [5–7]. Several phytobioactive substances such as curcumin, resveratrol, quercetin, epigallocatechin gallate, and berberine have been found to have anti-aging properties [5]. Thus, organismal development is the result of interactions between genes and epigenetic factors. Therefore, one of the main aims of current biomedical science and modern healthcare systems is to find a safe and effective dietary component of plant origin that has beneficial health effects or anti-aging properties for therapeutic purposes.

Turmeric (*Curcuma longa* L.), also known as Holud in Bengali, is generally utilized as a spice in curry and food additives. In the ethnomedicine of the Indian subcontinent, it has been used for centuries, for purposes such as improving memory and treating hypotonia and infertility in women, inflammation, and tumor- and age-related diseases: rheumatism, arthritis, diabetes, hypertension, and dementia [8–10]. Until now, at least 235 compounds from two major classes have been identified: polyphenolic curcuminoids and terpenoids volatile oil, including alkaloids and sterols [11]. The curcumin is the most abundant component of curcuminoids, whereas the principal components of volatile oil comprise α-turmerone, ar-turmerone, β-caryophyllene, β-turmerone, eucalyptol, and α-phellandrene. Numerous studies have reported the pharmacological properties of extracts, powders, oil, and curcumin. The extracts of curcuminoids have shown antioxidant, anti-inflammatory, anti-microbial, anti-cancer, anti-diabetic, anti-amyloidogenic, and neuroprotective activities [12, 13]. The concerted action of curcumin and demethoxycurcumin has been found to decrease lead-activated memorial loss in rats [14]. At the molecular genetic levels, turmeric or its components have been shown to influence several molecular targets that are involved in various biological processes such as cancer (p53, mTOR, Bcl2 and Bcl3, cyclin A and D, AP-1, AKt, PKc, NF-*k*B, Jak/Stat, and JNK), neurodegeneration (lipofuscin and Aβ deposition, lipid peroxidation, SOD and nrf2, COX2, LOX, and iNOS, Bax, and Bcl-xS), and aging (mTOR, DFF40, IAP, IIS, Aki, Bax and Bcl-xS, iNOS, NF-jB, LOX, and COX2) [15, 16]. Curcumin prolongs the lifespan and improves the health span of *Drosophila* in two ways: via regulating gene expression of the key enzymes SOD and CAT or reducing the expression of oxidative stress and age-related genes (*InR*, *TOR*, *hep*, *sun*, and *mth*) [17]. Curcumin also increases lifespan in a *Drosophila* model of Parkinson’s disease (PD) and promotes amyloid fibril conversion by reducing the pre-fibrillar/oligomeric species of amyloid β (Aβ). Additionally, curcumin blocks Aβ toxicity in vivo through the inhibition of Tau phosphorylation, resulting in reduced neurotoxicity in *Drosophila* [18–20]. Although curcumin has a wide spectrum of biological properties, factors such as low bioavailability, very poor absorption, rapid metabolism, and elimination of curcumin limit its therapeutic efficacy in humans [6, 21]. Shilpa Rawal et al. [22] have reported that turmeric increases the lifespan of *Drosophila* by scavenging the superoxide radicals by means of increasing the activity of total SOD and CAT and other unknown enzymes and target factors. However, this study did not evaluate the hormetic effect of turmeric on the lifespan of *Drosophila*, the many physiological processes of aging, and did not identify which unknown enzymes and target factors are involved. We therefore reviewed the possible affected protein in aging. It has been reported that the changes in tubulin are related to oxidative stress and age. Through in vitro polymerization experiments, it is evident that oxidative species drastically inhibited tubulin polymerization, a critical event for microtubule function. Peroxynitrite (ONOO_**¯**_), nitric oxide (NO), and nitroxyl donors progressively oxidize the cysteine thiol groups (SH) of tubulin monomers, thereby decreasing the ability of microtubules to polymerize [23]. It has been shown that tubulin protein in the human cerebral cortex decreased drastically with age [24, 25]. Recent studies report that the loss of α- and β-tubulin in the brain may occur due to excessive alcohol consumption and the natural aging of the brain [26]. Therefore, it can be reasonably assumed that the diet exerts an effect on tubulin protein level. How tubulin loss is associated with neurodegeneration and brain aging has been reported by in vitro and in vivo studies [27]. The knockdown of tubulin led to the aggregation of tau, which resulted in neuronal toxicity in *Caenorhabditis elegans*. However, suppression of tau in the tubulin knockdown model rescued neuronal function in *C. elegans*, while tubulin co-incubation inhibited tau aggregation in vitro. These results have linked tubulin loss to tau aggregation and tauopathy, a hallmark in neurodegeneration and normal brain aging. Therefore, halting tubulin protein loss in the brain should decrease molecular and cellular damage, delay the aging process, and eventually prolong lifespan.

Until now, no studies have stated the effect of turmeric or curcumin on housekeeping protein (especially tubulin) level in the brain and controlled for a number of physiological processes of aging. Therefore, we aimed to investigate the effect of dietary turmeric on the β-tubulin protein level and its relation with brain aging, as well as certain physiological traits, including survivability, locomotor activity, fertility, tolerance to oxidative stress, and eye health. In this respect, we took advantage of *D*. *melanogaster* as a model organism because of the relatively short lifecycle and simple culturing in the laboratory. Additionally, the similarities pertaining to the human aging process, as well as the genetics, metabolic pathways, nervous system, heart, kidney, and reproductive tract of mammals, render *Drosophila*, an effective model to investigate the basic knowledge on diverse gene-environment interactions and their connection to aging mechanisms [28, 29].

## Material and Methods

### *Drosophila* Culture Media Compositions, Chemicals Reagents, and Antibody

Standard cornmeal (DFC-30102), agar powder (DFY-30301), active dry yeast (HS-62–103), and p-hydroxy-benzoic acid methyl ester (DFM4-30,601) were from Hansol Tech, Seoul, Korea. RIPA buffer (89,900), halt protease inhibitor cocktail tablet (87,786), NuPAGE 4 × LDS sample buffer (NP0007), 4–12% Bis–Tris gradient gel (NP0329BOX), MES SDS running buffer (NP0002), chemiluminescent substrate, and Immun-Star™ WesternC™ Kit were purchased from Thermo Fischer Scientific, USA. Nitrocellulose membrane (1,620,112) and nonfat dry milk (1,706,404) were from Bio-Rad, USA. Curcumin (C1386-50G), BCA protein assay kit (QPBCA), Tween-20 (P2287-500ML), Triton X-100 (X100-500ML), DPPH (D9132), and rotenone (R8875) were bought from Sigma-Aldrich, Germany. Dithiothreitol (DTT) (M3278.0005) and β-mercaptoethanol (0482-100ML) were purchased from Genaxxon bioscience, Germany, and Amresco Inc., USA, respectively. Tris base (H5131) and glycine (G0709.1000) were from Promega Corporation, USA, and Duchefa Biochemie, Netherlands, respectively. L-Ascorbic acid (TC094) was collected from HiMedia, India. Primary mouse anti-β-tubulin monoclonal antibody (ab6046) and primary mouse anti-GAPDH monoclonal antibody (TA337137) were purchased from Abcam, UK, and OriGene Technologies, Inc., Rockville, MD 20,850, USA, respectively. The goat anti-mouse IgG (H + L) tagged with HRP is a secondary antibody obtained from Invitrogen Technology, USA. Propionic acid (163–04,726) and all other chemicals used in this study were from Wako Pure Chemical, Japan.

### Preparation of Turmeric Powder

The fresh rhizomes of turmeric (*Curcuma longa* L.) were collected from the field of Churamonkathi (7 m altitude above sea level), Jashore, Bangladesh, in March 2018. Botanical identification and authentication were made by a taxonomist, Dr. Sardar Nasiruddin, Chief Scientific Officer, National Herbarium, Dhaka, Bangladesh, where a Voucher number DACB 60,291 was deposited. The collected rhizomes were washed with tap water and sliced into a thin layer followed by drying at 25 °C in room air. The dried turmeric was blended rigorously to make a fine powder by a blender and stored in a clean and airtight container until used for media preparation.

### *Drosophila* Strain, Culture Maintenance, and Experimental Conditions

Isogenized *D*. *melanogaster* (*w1118*) was obtained from BDSC, Indiana University, Bloomington, USA, for use in this study. The *Drosophila* culture media (diet) was made as previously described with minor modifications [30]. A large number of *D*. *melanogaster* were cultured on standard corn meal-active dry yeast-sucrose media consisting of cornmeal (84 g), active dry yeast (24 g), sucrose (47 g), agar powder (8 g), 10% methyl-p-hydroxybenzoate in 95% ethyl alcohol (5 mL), and propionic acid (4 mL) in a total volume of 1 L. The culture was kept at 25 °C and 60–65% humidity during a 12-h dark/light cycle. For the control group, the food media was prepared without adding turmeric powder as a supplement. In the case of treatment, food media was prepared by mixing the regular food compositions with five different concentrations (0.125%, 0.25%, 0.5%, 1.0%, and 2.0%) of turmeric powder separately. The flies were shifted to a fresh diet every 2 days and, in some cases, every day to avoid media desiccation.

### Survival Assay

The *D*. *melanogaster* maintained on the regular diet at 25 °C were separated into six groups to distribute onto regular diet (control) and different concentrations (0.125%, 0.25%, 0.5%, 1%, and 2%) of turmeric powder-supplemented diet for releasing eggs, and the larvae were sustained in the respective diet at 25 °C until eclosion. In the 1–5-h time frame, the emergence of flies from the pupa was separated under brief CO_2_ anesthesia and kept in the respective diet at 25 °C for 24 h for maturation. A total of 1200 male and female flies were used for the survival assay. A total of 600 flies from each gender were distributed into a regular diet and five different concentrations of turmeric powder-supplemented diet. To avoid overcrowding, one hundred flies from each group were distributed into four separate vials containing the same diet (i.e., 25 flies per vial). Flies were then cultured at a constant temperature at 25 °C and humidity of 60–65% with a12-h dark/light cycle. Flies were transferred every day into new vials with fresh media to prevent non-age-associated mortality like getting stuck on food during the maintenance and culture, and their survivability was expressed. This procedure was continued until all flies were deceased.

### Climbing Assay

The climbing ability of flies was tested as previously described, with slight modifications [31]. Ten flies of each gender were examined for climbing ability as follows. Flies were tranquilized by CO_2_ and lodged into a blank plastic tube with a line-mark 4 cm from the bottommost of each tube. After a recovery period of about 30 min from the anesthetization, the *Drosophila* were gently tapped to the bottom of the tube, and the ability of each fly to climb 4 cm within 30 s was assessed. Flies crossing the 4-cm point within 30 s were counted as climbed flies, and the rest were considered unclimbed. This procedure was continued up to 40 days at 10-day intervals. The experiment was repeated three times with independently derived flies. The climbing performance index (PI) was determined for the individual diet-fed group of *Drosophila* by using the equation: PI = 0.5 × (*c*_total_ + *c*_top_ –*c*_bottom_)/c_total_, where *c*_total_ is the full count of *Drosophila*, *c*_top_ is the full count of *Drosophila* above line-mark 4 cm of the tube, and *c*_bottom_ is the full count of *Drosophila* under the line-mark 4 cm of the tube.

### Fertility Assay

The fertility test was conducted as per the procedure designed by Tain et al. [32]. To evaluate the effect of turmeric dose on fertility, six sets of flies, each set with a single 1-day-old male and three virgin females, were placed into a vial containing a regular diet and five separate vials, each containing a different concentration of turmeric powder-supplemented diet. After 5 days of mating, adult flies were taken out from the culture vials, and the total abundance of larvae was counted from each vial by separating the larvae in a 10% sucrose solution [33]. Among the five concentrations of turmeric, 0.5% showed the best result in emerging larvae. Therefore, the 0.5% turmeric powder-supplemented diet was used to evaluate the effect of turmeric on the fertility of the aging progeny at the ages of 5th, 10th, 20th, 30th, 45th, and 55th days. To do this, every time two sets of flies, each with a single age-matched male with age-matched 3 virgin females, were placed either into a regular diet or a 0.5% turmeric powder-supplemented diet for mating. After 5 days of mating, the total number of larvae was counted from each vial with a 10% sucrose solution. Flies were nurtured at 25 °C and 60–65% humidity with a 12-h dark/light cycle.

### Preparation of *D. melanogaster* Brain Homogenates

The *Drosophila* brain homogenates were prepared as previously described, with minor modifications [34]. Briefly, a total of ten heads were dissected from age-matched flies raised on a regular diet or one of various concentrations of turmeric powder-supplemented diet and kept into individual Eppendorf tubes at 4 °C. The appropriate number of zirconium beads and 30 μL of RIPA buffer containing halt protease inhibitor cocktail were added to each Eppendorf tube. Fly heads were homogenized by Bullet Blender Tissue Homogenizer (Blue BBX24B, Next Advance Inc., USA) three times at 7 rpm, each with 2 min. After that, the samples were centrifuged for 5 min at 13,000 rpm and 4 °C. The supernatant was collected and centrifuged again under the same conditions. The fresh supernatant was used for all experiments. The quantity of protein of head homogenates was measured by using the BCA protein test kit.

### Western Blot Analysis

The same amount of head homogenate was added with 4 × LDS sample buffer and 100 mM of DTT to an ultimate density of 10 mM in a total of 16 μL sample solution. The sample solutions were heated at 95 °C for 6 min, and 10 μL of heated sample was loaded into each well of Bis–Tris gradient gel (4–12%) followed by electrophoresis in MES SDS running buffer. The protein was then electrotransferred from SDS-PAGE gel to an NC membrane in the Tris–Glycine transport buffer. Five percent nonfat dry milk in PBST-Triton X-100 was used to block the NC membrane for 1 h at RT. The NC membrane was immersed in PBST-Triton X-100 for 2 min followed by incubation overnight at 4 °C in mouse anti-β-tubulin (1:5000) monoclonal antibody with gentle rocking. Following 3 × 10 min washing with PBST-Triton X-100, the membranes were developed for 2 h at RT with goat anti-mouse IgG (H + L) tagged with HRP diluted at 1:2500. Following washing, Immun-Star™ Western^C^™ Kit, the chemiluminescent substrate was applied to the NC membrane, and pictures were taken with a Fusion-FX7-spectra equipped with a CCD camera (Vilber lourmat, France). After detection of β-tubulin, the membranes were washed in PBST-Triton X-100 for 5 min and transferred to the pre-warmed de-staining solution and incubated for 45 min at 60 °C with gentle rocking at 80 rpm. The membrane was then washed with distilled water and PBST-Triton X-100, each for 3 min, and blocked with 5% skimmed milk in PBST-Triton X-100 for 2 h. After 2 min washing with PBST-Triton X-100, the membrane was incubated overnight at 4 °C in primary mouse anti-GAPDH monoclonal antibody (1:2500) with gentle rocking. Subsequent steps were performed as described above. The primary and secondary antibodies were properly mixed with PBST-Triton X-100 containing 1% skimmed milk.

### Oxidative Stress Assay

The oxidative stress assay was carried out by following the method described by Hosamani et al. with minor modifications [35]. Briefly, to see the effect of turmeric on rotenone-induced oxidative stress, the method employed for the survival assay was redone for the oxidative stress assay. Different concentrations of rotenone (250 μM, 500 μM, and 1000 μM) were added as a supplement in a regular diet and in a diet with 0.5% turmeric. A total of 1200 male and female flies were used in this assay. For each gender, a total of 600 flies raised in regular diet were distributed into three groups of regular diet (regular diet + 250 μM rotenone, regular diet + 500 μM rotenone, and regular diet + 1000 μM rotenone) and three groups of turmeric powder-supplemented diet (turmeric powder-supplemented diet + 250 μM rotenone, turmeric powder-supplemented diet + 500 μM rotenone, and turmeric powder-supplemented diet + 1000 μM rotenone). To avoid overcrowding, one hundred flies of each group were distributed into five separate vials containing the same diet (i.e., 20 flies per vial). Flies were then cultured at a constant temperature of 25 °C and a humidity of 60–65% with a 12-h dark/light cycle. During this assay, flies were transferred every day into new vials with a fresh diet, and their survivability was evaluated. This procedure was carried out until all flies were deceased.

### Antioxidant Activity Test

The antioxidant potentiality of turmeric extracts was ascertained by means of a DPPH-free radical-capturing activity assay, as previously described by Braca and colleagues, with slight modifications [36]. The stock concentrations (1.5 mg/mL) of test samples, ascorbic acid, and DPPH were prepared in methanol. Sixty microliters of 1.5 mg/mL DPPH were added to various concentrations (1.75 to 112 μg/mL) of test samples and ascorbic acid to a final volume of 3.0 mL separately. The positive control consisted of the various concentrations of ascorbic acid, and the negative control was a 3.0 mL solution of 30 μg/mL DPPH in methanol. The content of each preparation was appropriately mixed and stowed in the dark for 10 min at RT. The optical density was measured at 517 nm in a UV spectrophotometer (V-1100, Mapada Instruments Co. Ltd., Shanghai). The tests of the samples were repeated three times. The percent of DPPH inhibition was assessed by means of the following formula:

$$Percent\;\left(\%\right)\;of\;DPPH\;inhibition=\left[\left(\frac{A_{DPPH}-A_S}{A_{DPPH}}\right)\right]\times100$$

### Light Microscopic Observation of *D*.* melanogaster* Eye

The light microscopic observation of the *Drosophila* eye was performed as previously described, with slight modifications [30]. Freshly collected virgin flies of both genders were supplied with the appropriate diet and maintained as in the survival assay. Flies of the appropriate ages and genders from each respective diet were killed by carbon dioxide anesthesia and kept in an Eppendorf tube. The *Drosophila* were frozen at − 80 °C for 1 h and attached on their edges and observed under a Stereo Microscope of Greenough Optical System (EZ4, Leica Microsystem Inc., Germany). Photographs were obtained at the highest magnification (35 ×).

### High-Performance Liquid Chromatography Analysis

For the quantification of curcumin in five different concentrations of turmeric powder, we did high-performance liquid chromatography (HPLC) as the previously described, with minor modifications [37]. HPLC was carried out by a Shimadzu-VP system from Japan. A C18 column flow ratio of 1.0 mL/min and a PDA indicator used with a wavelength of 425 nm were employed. The sample (20 μL) was injected into the column, and the temperature was maintained at 40 °C. The HPLC column was cleansed by filtration, degassing, and elution. Elution was done for 1 h, followed by washing the column for 1 h. The mobile phase solution was prepared by the 50:1:49 combinations of acetonitrile, acetic acid, and aquabides. Following washing, the column was equilibrated by eluting the mobile phase solvent for 30 min. 2.5 mg of standard curcumin was appropriately measured into a volumetric flask and resolved in the mobile phase solution with warming, and this was used as the stock solution of curcumin. The working solution of different concentrations of curcumin was prepared by taking aliquots of stock solution of curcumin for the calibration plot and injected. The standard solutions of curcumin at the concentrations of 5, 7.5, 10, 12.5, and 15 ppm were prepared for obtaining a linear curve. Turmeric sample preparation was based on several concentrations of turmeric powder (0.125%, 0.25% 0.5%, 1.0%, and 2.0%), each of which was prepared in an individual volumetric flask. The turmeric solutions were sonicated for 10 min, filtered, and then diluted 100 times with a mobile phase solution. Every solution was filtered with a 0.45-μM syringe filter before injection into the HPLC column. The standardized solution was loaded into the injector once every time, and the dimension of the curve was noted and enumerated for the *R*-value and *R*^2^. The correlation coefficient of simple linear regression calculated by the equation is *y* = *ax* + *b*.

### Statistical Analysis

The experimental data were represented as the average ± STDEV of two replicates for Western blot and three replicates for other tests. The survivability of cohorts was compared using a Log rank test for survival curves comparisons and Fisher’s exact test with Bonferroni corrections to estimate differences in survival percentiles and medial lifespan. The statistical interpretation of the other tests of three replicates was accomplished using one-way ANOVA by Origin Lab version 2018, and the Bonferroni and Tukey post-hoc tests were used for these tests. *p < 0.05, **p < 0.01, and ***p < 0.001 are positively and ^#^*p* < 0.05, ^##^*p* < 0.01, and ^###^*p* < 0.001 are negatively significant in comparison to control values.

## Results

### Dietary Turmeric Exerted a Hormetic Effect and Prolonged the Lifetime of *D*. *melanogaster*

**Fig. 1:**
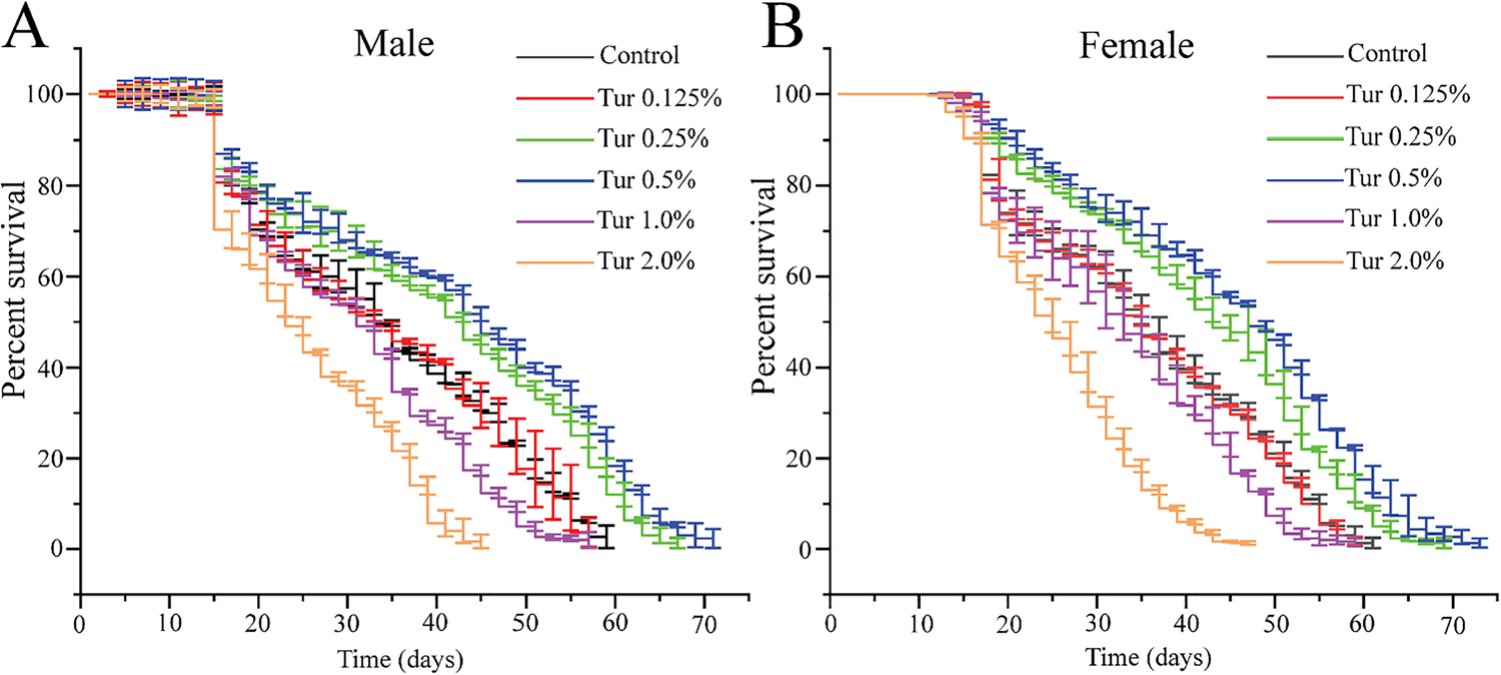
Survival curves for flies fed a control diet versus five different concentrations of turmeric-supplemented diets. **A** *w1118* male and **B** *w1118* female. Control denotes the regular diet (standard cornmeal media), and Tur denotes the groups of the regular diet were supplemented with 0.125%, 0.25%, 0.5%, 1.0%, and 2.0% turmeric powder. Each curve depicts the percentage of living flies during a given period of time. The data were from three separate studies, and values represented as average ± SE. The survivability of cohorts was compared using a Log rank test for survival curves comparisons and Fisher’s exact test with Bonferroni corrections to estimate differences in survival percentiles and median lifespan (Supplementary Table S3 for complete statistics)

To assess the effectiveness of turmeric on lifespan, *Drosophila* were fed either a regular diet or one of five different concentrations of turmeric powder-supplemented diet (Table S1). The effect of turmeric on lifespan was estimated by counting the surviving flies upon age progression. As shown in Fig. 1 and Supplementary Table S2, the degree of variation in survivability between each group of feeding regimens became clear from the 15th day. When *Drosophila* was fed only a regular diet, male *Drosophila* died at 57–59 days and female *Drosophila* died at 59–61 days. With regard to *Drosophila* fed different concentrations of turmeric-supplemented diet, there was no increase in lifespan at 0.125% but a moderate increase in the lifespan of male (66–67 days) and female (67–69 days) *Drosophila* at 0.25% turmeric powder-supplemented diet. With the increased dose of turmeric, there was a remarkable increase in the lifespan of male (69–71 days) and female (71–73 days) *Drosophila* up to a concentration of 0.5%. Beyond that, the lifespan of male and female *Drosophila* gradually decreased to 55–57 days and 57–59 days, respectively, at a 1% turmeric-supplemented diet and 43–45 days for males and 45–47 days for females at a 2% turmeric-supplemented diet. However, the median lifespan of male and female *Drosophila* (Table S3), at the turmeric concentration of 0.25% and 0.5%, was significantly (Log Rank test, *p* < 0.001) increased compared to the regular diet-fed group. In contrast, the median lifespan of both the male and female flies, at the turmeric concentration of 1.0% and 2.0%, was significantly (Log rank test, *p* < 0.001) decreased compared to the control group. Overall, a 0.25% turmeric-supplemented diet extended the lifespan by 24% in male and female *D. melanogaster*. A 0.5% turmeric-supplemented diet increased the lifespan by 24% in males and 35% in female *D. melanogaster*. Regrettably, 1.0% turmeric-supplemented diet decreased the lifespan by 12% in males and female *D. melanogaster*. Collectively, these results indicate the hormetic influence of turmeric on the lifespan of *D. melanogaster* and also implicate that 0.5% turmeric is an optimal dose with potential life-extending properties, as well as eliciting desirable effects in aspects of other physiological processes.

### Dietary Turmeric Exhibited a Hormetic Effect and Ameliorated the Locomotive Decrease of *D*.* melanogaster* Towards Elderliness

**Fig. 2:**
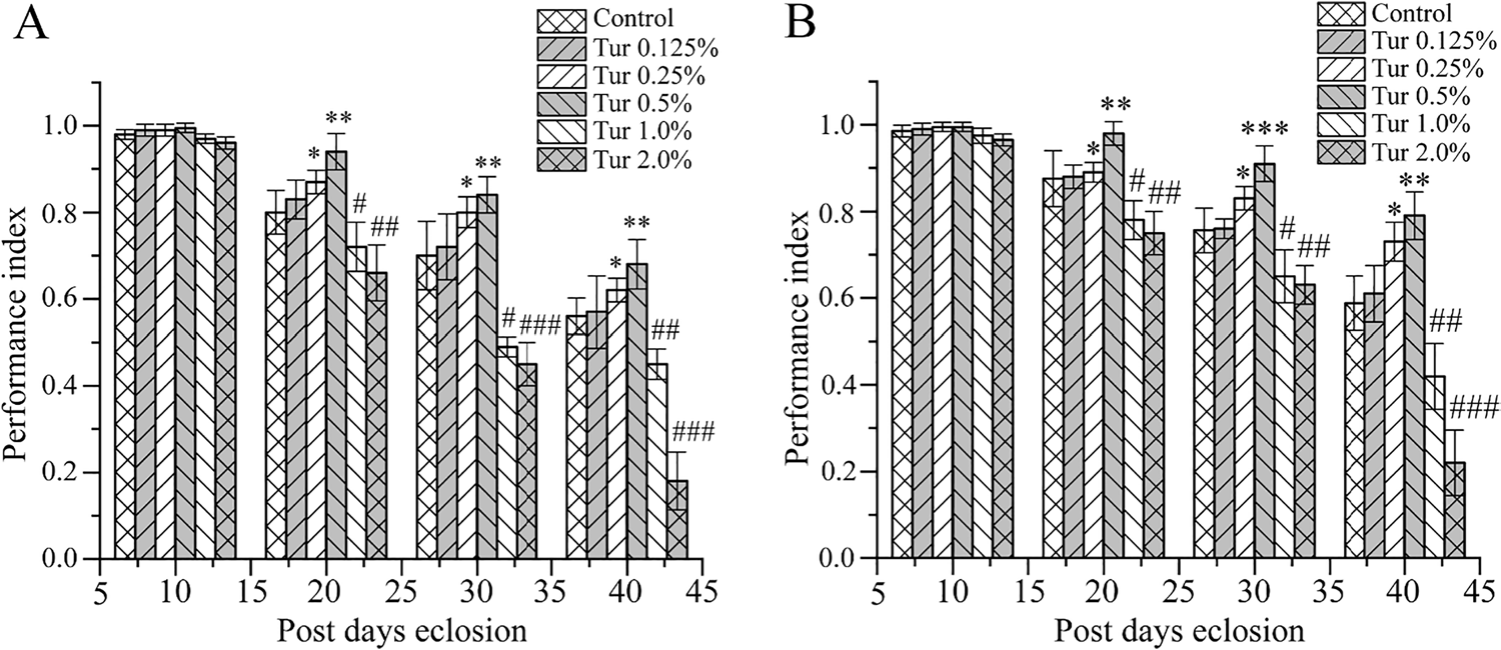
The dietary turmeric prevented age-induced locomotion at the lower doses but aggravated age-induced locomotion at the higher doses. **A** *w1118* male and **B** *w1118* female. Control designates the regular diet (standard cornmeal media) fed *Drosophila*, and Tur represents turmeric powder-supplemented diet-fed *Drosophila*. The locomotive performance was monitored on the 10th, 20th, 30th, and 40th day. The statistical difference between control and turmeric-supplemented diet groups was interpreted through Tukey and Bonferroni post-hoc analyses following one-way ANOVA. Ten flies were taken from each feeding condition for climbing assay. The experiment repeated three times with independently derived flies to get the mean number. Values were represented as average ± STDEV. *p < 0.05, **p < 0.01, and ***p < 0.001 are positively significant related to the corresponding control (regular diet). #p < 0.05, ##p < 0.01 and ###p < 0.001 are negatively significant related to the respective control (regular diet)

The locomotion performance assay aims to assess the muscle function, as muscular integrity is indispensable for proper locomotion. Therefore, the decrease in locomotion is a key characteristic of aging [30]. We thus explored the locomotive performance of *D. melanogaster* by determining the climbing performance up to 40 days of age in 10-day intervals (Fig. 2A and B). Flies in the control group and turmeric-supplemented group exhibited no significant difference in locomotion up to 10 days. The locomotive difference was observed on the 20th day in both male and female *D. melanogaster* in the control and turmeric-supplemented diet groups. Although the flies in the control and turmeric-supplemented diet groups displayed a gradual decrease in mobility with age, the decline of locomotion was significantly lower in 0.25% (*p* < 0.5) and 0.5% (*p* < 0.01) turmeric powder-supplemented diet groups than that of control groups. Conversely, 1.0% and 2.0% turmeric-supplemented diet groups of both male and female *Drosophila* showed a significant deterioration in locomotive ability instead of improving the locomotor activities compared to control. These findings again indicate the hormetic influence of turmeric with regard to locomotion in *D. melanogaster* and also imply that 0.5% turmeric is an optimal dose that significantly ameliorated the locomotive deterioration of *D. melanogaster* with age; this is in line with the lifespan extending results described above (Fig. 1A and B).

### Dietary Turmeric Exhibited a Hormetic Effect: the Optimal Doses Caused Improved Fertility and Higher Doses Declined Fertility

**Fig. 3:**
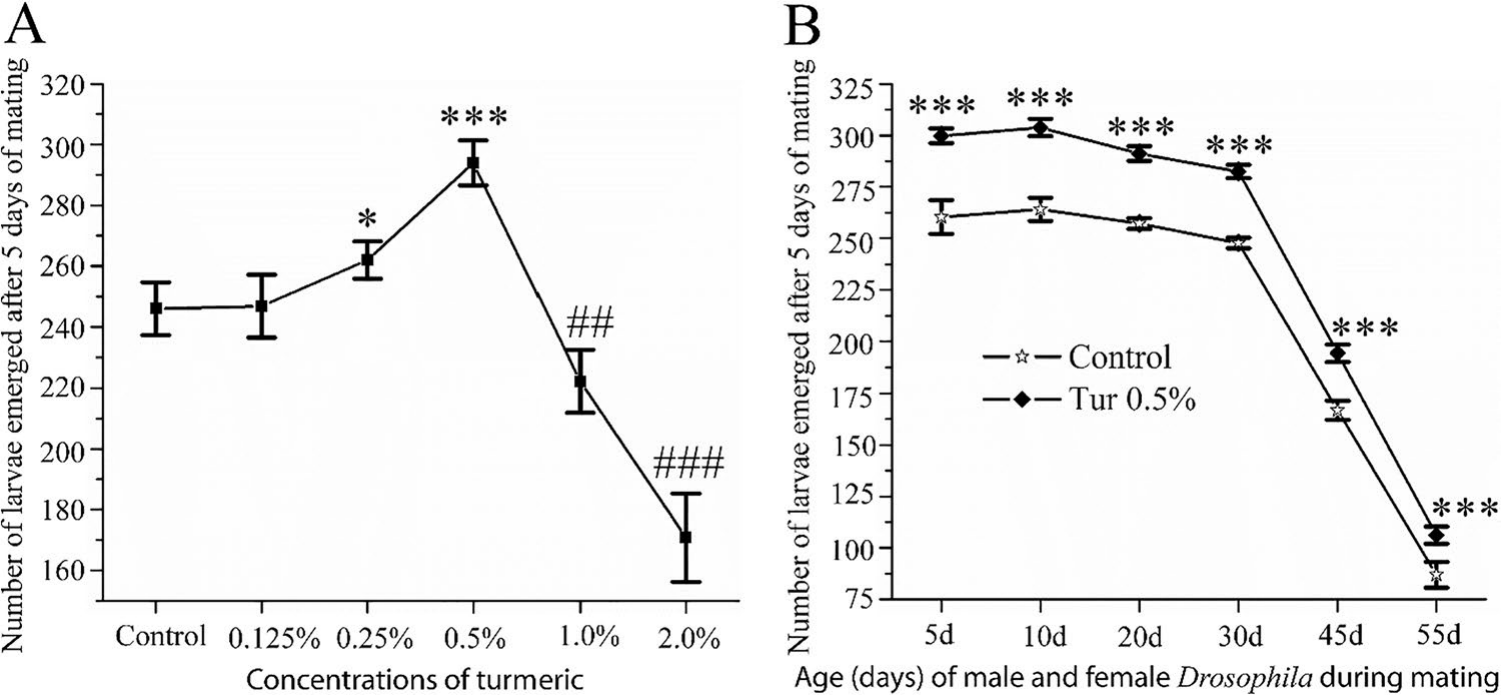
Hormetic consequence of turmeric on the fertility of *D*. *melanogaster*. **A** The lower doses of turmeric improved the fertility of *D*. *melanogaster*, but the higher doses deteriorated fertility. Virgin male and female *D. melanogaster* at 1 day of age were crossed on a regular diet and various concentrations of turmeric-supplemented diet. *p < 0.05 and ***p < 0.001 are positively and ^##^*p* < 0.01 and ^###^*p* < 0.001 are negatively significant related to the corresponding control (regular diet). **B** The optimal dose, 0.5% turmeric, supplemented diet improved the fertility of *D*. *melanogaster* upon age progression of different adult stages of *Drosophila* compared to *Drosophila* mated in a regular diet. Virgin male and female *D. melanogaster* at the ages of day 1, 10, 20, 30, 45, and 55 were mated on a regular diet and only 0.5% of the turmeric-supplemented diet. ***p < 0.001 is statistically significant when compared to the corresponding control (regular diet), because gradually aging flies mated in a 0.5% turmeric-supplemented diet had higher fertility than gradually aging flies crossed on a regular diet. **A**–**B** The statistical difference between control and turmeric-supplemented diet groups was interpreted through Tukey and Bonferroni post-hoc analyses following one-way ANOVA. The experiment repeated three times with independently derived flies to get the mean number. Values were represented as average ± STDEV

The link between age progression and low fertility is widely observed across the tree of life [38]. We, therefore, carried out fertility assay in two ways: (i) mating between single 1-day-old male and three virgin females together in different concentrations of turmeric-supplemented diet and regular diet and (ii) mating between male and female flies at the age of 1, 10, 20, 30, 45, and 55 days in only 0.5% turmeric-supplemented diet and regular diet. As shown in Fig. 3A, when *Drosophila* were fed turmeric-supplemented diet, there was no increase in the number of larvae at 0.125% turmeric-supplemented diet, but a significant increase in the number of larvae (*p < 0.05) at 0.25% turmeric-supplemented diet were counted compared to the regular diet. With the increased dose of turmeric, there was a remarkable increase in the number of larvae until a point of turmeric concentration (0.5%) (***p < 0.001); beyond that, the larvae count significantly decreased at 1% (^##^*p* < 0.01) and 2% (^###^*p* < 0.001) turmeric-supplemented diet. These findings highlight the hormetic influence of turmeric on the fertility of *D. melanogaster* and also suggest that 0.5% turmeric is an optimal dose for improving the fertility of *D. melanogaster*. To determine whether turmeric could ameliorate the age-induced declined fertility, the fertility of gradual aging flies at the age of 1, 10, 20, 30, 45, and 55 days was assessed by mating age-matched male and female flies in 0.5% turmeric-supplemented diet and regular diet. The number of emerged larvae in each of the turmeric-supplemented diets and regular diet was recorded after 5 days of mating. The elderliness of the flies significantly affected their fecundity in that the fertility of flies dropped with age (Fig. 3B). However, supplementation of 0.5% turmeric with a regular diet significantly (***p < 0.001) improved the fertility of the flies. Above all, 10-day-old flies had higher fertility than all other age groups of flies.

### The Hormetic Effect of Dietary Turmeric: the Optimal Concentration Improved β-Tubulin Protein Level and Higher Doses Decreased β-Tubulin Protein Level in the ***Drosophila*** Brain

**Fig. 4:**
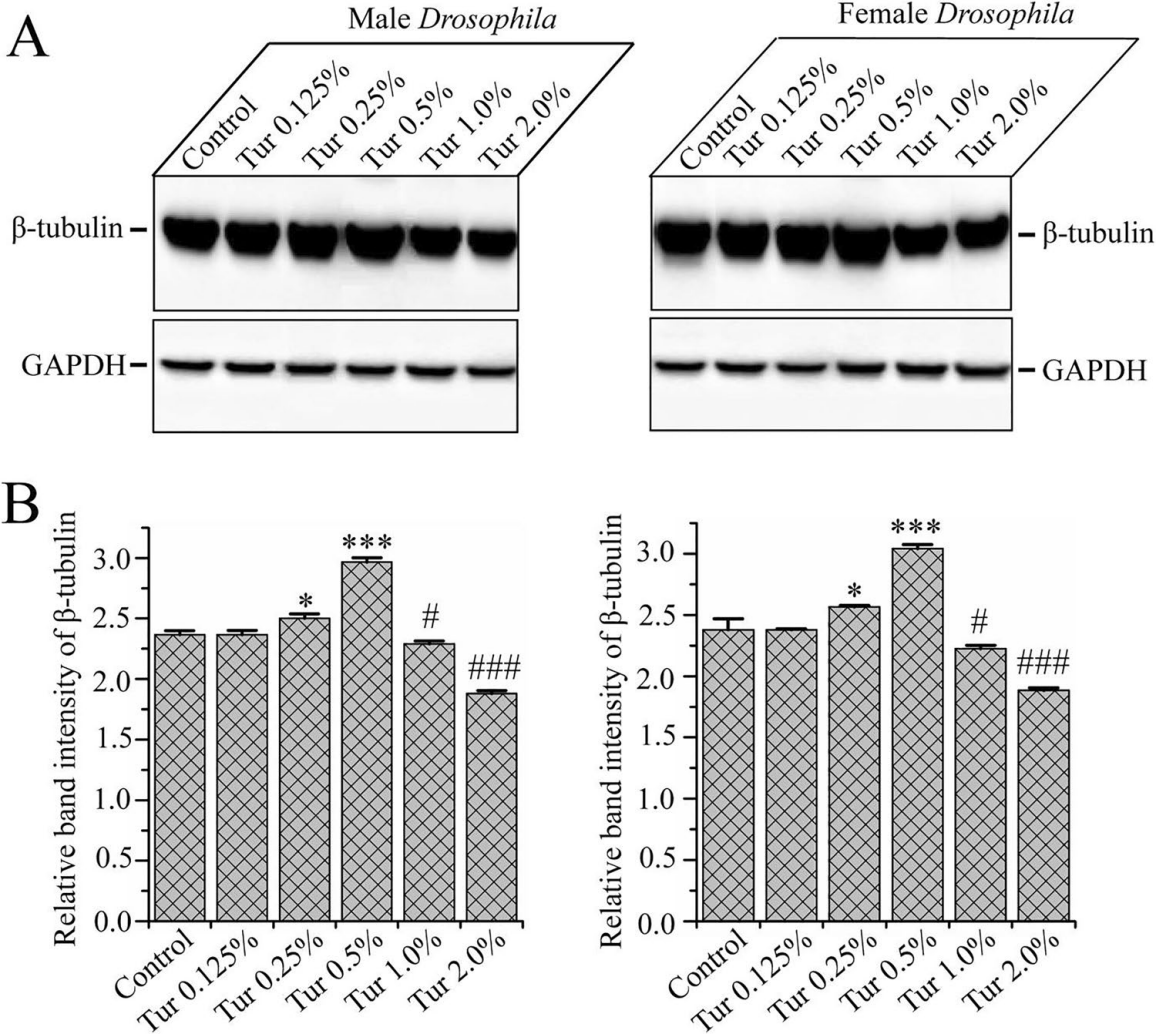
The biphasic effect of the doses of turmeric on β-tubulin protein level. **A** The β-tubulin level in both gender of *Drosophila* (upper panel) gradually increased as the turmeric concentration increased up to 0.5%, and the highest level of β-tubulin observed at 0.5% turmeric-supplemented diet. The β-tubulin level was decreased gradually at the concentrations of 1% and 2% turmeric. The level of tubulin observed on the 30th day and determined by rabbit anti-β-tubulin primary antibody. GAPDH as loading control showed in the lower panel. There was no major discrepancy in GAPDH level in both gender of *Drosophila* reared on a regular diet and 0.125%, 0.25%, 0.5%, 1.0%, and 2.0% turmeric powder-supplemented diets. **B** The relative level of tubulin and GAPDH calculated densitometrically. The data represented as the proportion of β-tubulin band intensity to the GAPDH band intensity. The experiment repeated three times with independently derived flies to get the mean number. Values were represented as average ± STDEV. The statistical difference between control and turmeric-supplemented diet groups were interpreted through Tukey and Bonferroni post-hoc analyses following one-way ANOVA

Tubulin protein is the building block of the microtubule filament and is a key component of the cytoskeleton. It is required for neuronal contour and stabilization, axon and dendrite elongation, muscular strength, and connections for cellular transportation and cell division [39]. Therefore, we analyzed β-tubulin protein level in the brain of the 30-day-old cohort of *D. melanogaster* cultured on either regular diet or various concentrations of turmeric-supplemented diet. Western blot data (Fig. 4A, B and Supplementary Figure S1) exhibited that β-tubulin protein level was unchanged at 0.125% turmeric. However, the β-tubulin protein level was gradually increased with an increase in turmeric concentration up to 0.5% (*p* < 0.001) and significantly decreased as the level of turmeric increased to 1.0% (*p* < 0.05) and 2% (*p* < 0.001) turmeric. However, no changes were observed in the levels of GAPDH across the entire lifespan of the tested *Drosophila.* Our Western blot results suggest that turmeric has a dual role that is attributed by its concentrations, a stimulating effect on β-tubulin levels at the optimal concentrations, while higher concentrations are found to have an inhibitory effect.

### Βeta-Tubulin Protein Level Decreased with Age in *D*.* melanogaster*

**Fig. 5:**
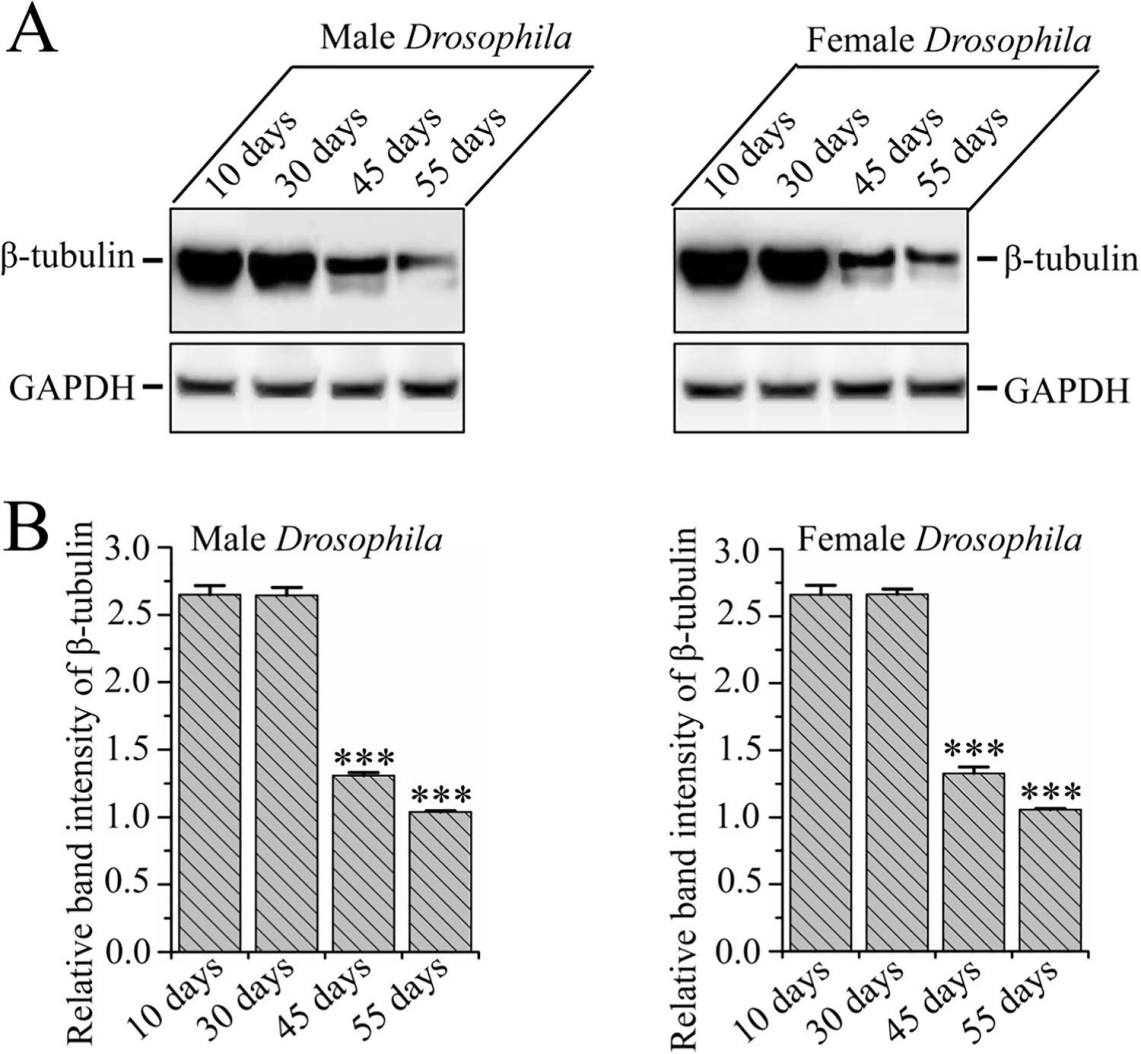
The pattern of age-related β-tubulin level across the lifespan of *D. melanogaster*. **A** β-tubulin protein level in male and female *D. melanogaster* cultured in a regular diet. There was no difference observed for GAPDH level entire the lifespan of both gender of *D. melanogaster*. **B** The relative level of β-tubulin and GAPDH ascertained densitometrically. The data were represented as the proportion of desired protein intensity to the GAPDH intensity. The experiment was repeated three times with independently derived flies to get the mean number. Values were represented as average ± STDEV. The statistical difference was compared with a 10-day-old fly with other older flies and interpreted through Tukey and Bonferroni post-hoc analyses following one-way ANOVA

It has been reported that cytosolic tubulin proteins declined with age in the brain, which causes altered neuronal structure and deterioration of interneuronal connections, giving rise to deficiencies of brain functions that have consequential effects on the neuropathology of aging in humans [26, 27]. Therefore, we checked the level of β-tubulin protein in the brain of *D. melanogaster* over time. Western blot data showed that there was no significant change of β-tubulin level up to the age at 30 days. However, after 30 days, a significant decrease in β-tubulin levels was observed. This continued with increasing age (Fig. 5A and B). Interestingly, no change was observed in GAPDH levels across the entire lifespan of *D*. *melanogaster*. Our Western blot results showcase that the level of β-tubulin protein decreased in the aging *D. melanogaster* brain, similarly to the depletion of tubulin protein in the aging human brain; this is consistent with the lifespan, locomotion, and fertility decrement data (Figs. 1, 2, and 3).

### Turmeric Ameliorated the Aging-Induced Loss of β-Tubulin Protein in *D*. *melanogaster* Brain

**Fig. 6:**
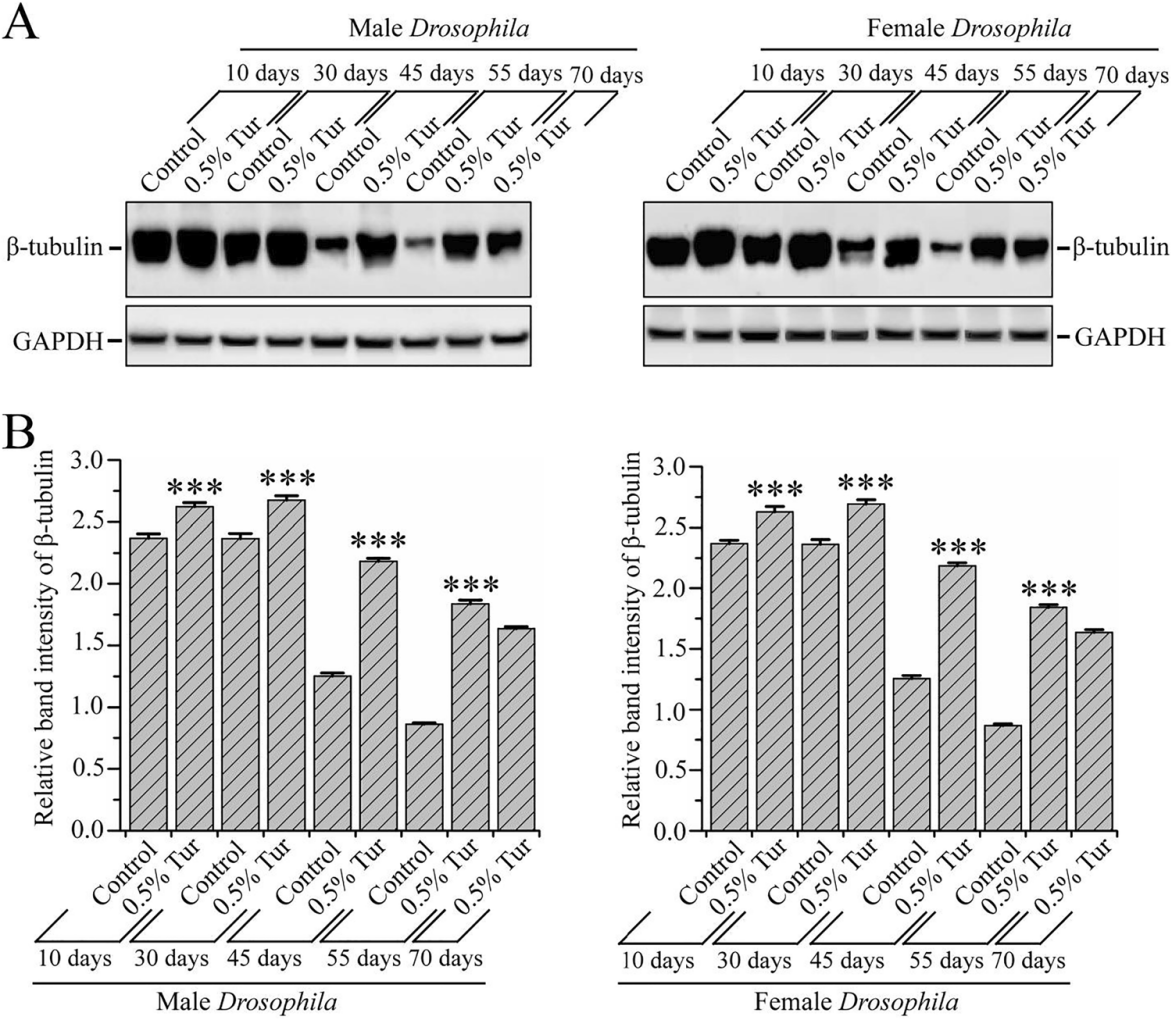
Turmeric remedied the aging-associated decline of β-tubulin protein level in *D. melanogaster* brain*.* **A** Beta-tubulin protein level in male and female *D. melanogaster* reared in control diet and 0.5% turmeric-supplemented diet. No significant discrepancy in GAPDH level was observed in the whole lifespan of both gender of *D. melanogaster*. **B** The relative level of tubulin and GAPDH computed densitometrically; the data were represented as the ratio of β-tubulin band intensity to the GAPDH band intensity. The experiment repeated three times with independently derived flies to get the mean number. Values were represented as average ± STDEV. The statistical difference between control and turmeric-supplemented diet groups was interpreted through Tukey and Bonferroni post-hoc analyses following one-way ANOVA

Since the 0.5% turmeric supplementation diet improved the β-tubulin protein level in the *Drosophila* brain (Fig. 4), we cultured *Drosophila* in a regular diet and 0.5% turmeric-supplemented diet until their death to see the effect of turmeric on β-tubulin protein level in the brain of *Drosophila*. As shown in Fig. 6A and B, there was no significant change in β-tubulin protein level in regular diet-fed *Drosophila* up to 30 days. But a decrease in β-tubulin protein level started after 30 days, and this was continued with increasing the age in both genders of *D. melanogaster* cultured on regular diet. Conversely, the level of β-tubulin protein level was significantly (*p* < 0.001) increased by 24.32% in males and 22.69% in females of 10-day-old *Drosophila* and 26.59% in males and 24.08% in females of 30-day-old *Drosophila* cultured in 0.5% turmeric-supplemented diet. At the age of 45 and 55 days, the β-tubulin protein level was significantly decreased both in male and female *Drosophila* cultured in a regular diet. However, it is interesting that this aging-induced decrement of β-tubulin protein level is ameliorated both in male and female *Drosophila* reared in a 0.5% turmeric-supplemented diet. Not only that, but both the male and female *Drosophila* cultured in a 0.5% turmeric-supplemented diet also survived for up to 70 to 75 days, and these flies were found to show higher levels of β-tubulin protein level. This result indicates that an elevated level of β-tubulin protein is connected with longevity of *D. melanogaster*.

### Turmeric Suppressed Oxidative Stress-Triggered Lethality in *D*.* melanogaster*

**Fig. 7:**
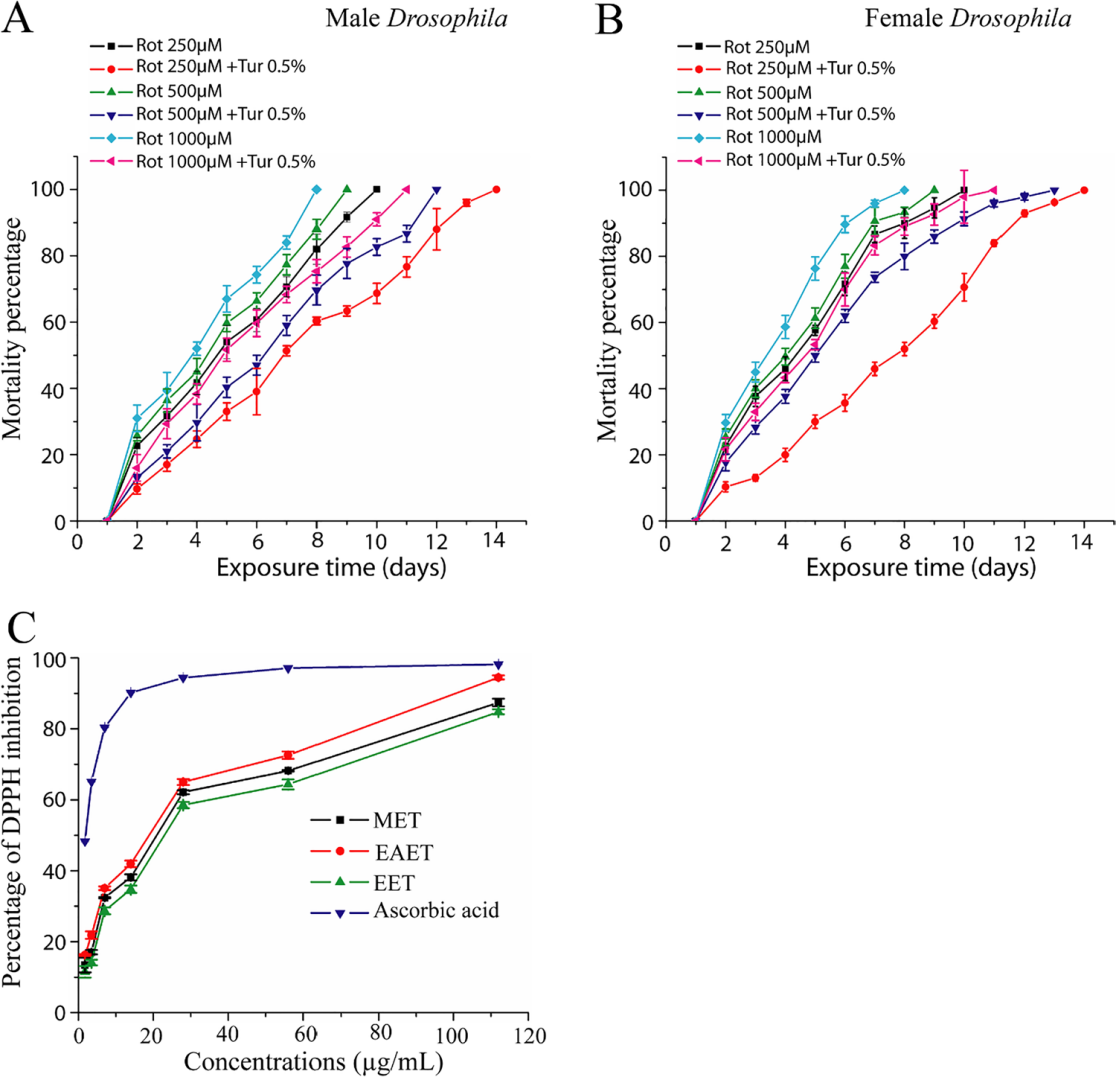
Rotenone-triggered lethality in *D. melanogaster* and its suppression by antioxidative properties of turmeric. Mortality percent among male (**A**) and female (**B**) *Drosophila* cultured to rotenone (250, 500, and 1000 μM)-supplemented diet and rotenone with 0.5% turmeric-supplemented diet. Rotenone appearance posed a concentration-reliant lethality both in male and female *Drosophila*, and this lethality reduced when *Drosophila* exposed to a 0.5% turmeric-supplemented diet. **C** Percentage of DPPH-free radicals inhibition by methanol, ethyl acetate, and ethanol extracts of turmeric. MET, methanol extract of turmeric; EAET, ethyl acetate extract of turmeric; EET, ethanol extract of turmeric

Rotenone is a blocker of complex I in the mitochondrial respiratory chain, causing the increased development of ROS, which enhances mortality [40]. Therefore, we hypothesize that if dietary turmeric gives the defense against rotenone-induced lethality, it would be a life-extending bioactive agent. As shown in Fig. 7A and B, exposure of both male and female flies to rotenone, and rotenone with 0.5% turmeric resulted in a rotenone concentration-dependent lethality over a 14-day experimental period. One hundred percent mortality of flies occurred at 8, 9, and 10 days and 11, 12, and 14 days when flies were cultured in 1000, 500, and 250 μM rotenone-supplemented diet and 1000, 500, and 250 μM rotenone with 0.5% turmeric-supplemented diet, respectively. These data showed that rotenone causes a higher incidence of mortality, which was reduced by turmeric, indicating that turmeric acts as an anti-reactive oxygen species (ROS) agent for reducing rotenone-induced mortality of *Drosophila*. Since turmeric reduced rotenone-induced mortality, we investigated whether turmeric can scavenge reactive oxygen species (free radicals). The data showed that ethanol, methyl alcohol, and ethyl acetate extracts of turmeric at the concentration of 1.75, 3.5, 7.0, 14.0, 28.0, 56, and 112 μg/mL have dose-dependent DPPH-free radical scavenging capacity (Fig. 7C). The ethyl acetate, methanol, and ethanol extracts displayed antioxidant activity ranging between 16.24 ± 0.240 and 98.98 ± 0.243%, 13.41 ± 2.050 and 95.949 ± 0.1575%, and 11.487 ± 1.630 and 94.735 ± 0.243%, respectively. The positive control, ascorbic acid, exhibited antioxidant activity with the range between 48.316 ± 0.165 and 99.251 ± 0.093%. The ranking of the free radical entrapping capability of ethyl acetate, methyl alcohol, and ethyl alcohol extracts of turmeric was EAET > MET > EET. This result suggests that turmeric might have bioactive compounds responsible for neutralizing rotenone-induced oxidative stress.

### Turmeric Suppressed Age-Related Eye Deterioration in *D. melanogaster*

**Fig. 8:**
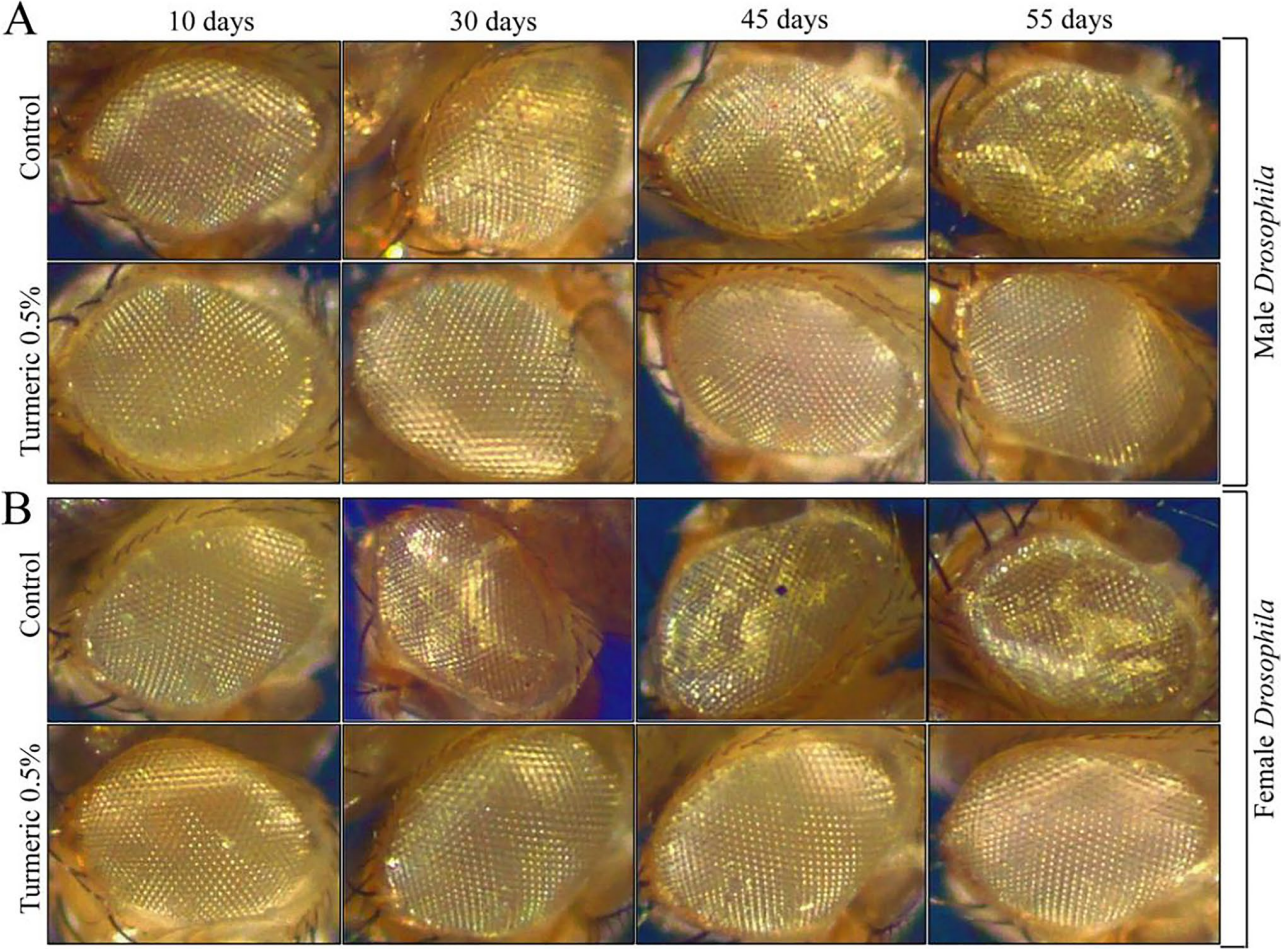
Light microscopy observation of the *Drosophila* compound eyes during age progression. Turmeric prevented the developmental eye defects in male (**A**) and female (**B**) *D. melanogaster* with age progression. Control represents regular diet, and Tur represents regular diet supplemented with 0.5% turmeric powder. Light microscopic observation of *Drosophila* eye done on 10, 30, 45, and 55 days post-eclosion

The eyes of *Drosophila* are well recognized as a typical model for depicting the combined effects of neurodegeneration and aging [30]. Thus, we assessed the phenotypical appearance of the eye with age progression. As shown in Fig. 8A and B, the phenotypical appearance of the *Drosophila* eye strongly exhibited the progressive damage (eye lesions and loss of ommatidial structure) that begin from the 30th day of age in the regular diet-fed *Drosophila* with degeneration increasing with age. On the contrary, flies fed the turmeric-supplemented diet showed reduced eye deterioration upon age progression. Remarkably, the eyes of both the male and female flies raised on a turmeric-supplemented diet even at the 55th day of age were considerably unlike those of the regular diet-fed groups of flies. Taken together, it has been concluded that turmeric ameliorates eye degeneration during age progression.

### Quantification of Curcumin Content in Turmeric Powder

**Fig. 9:**
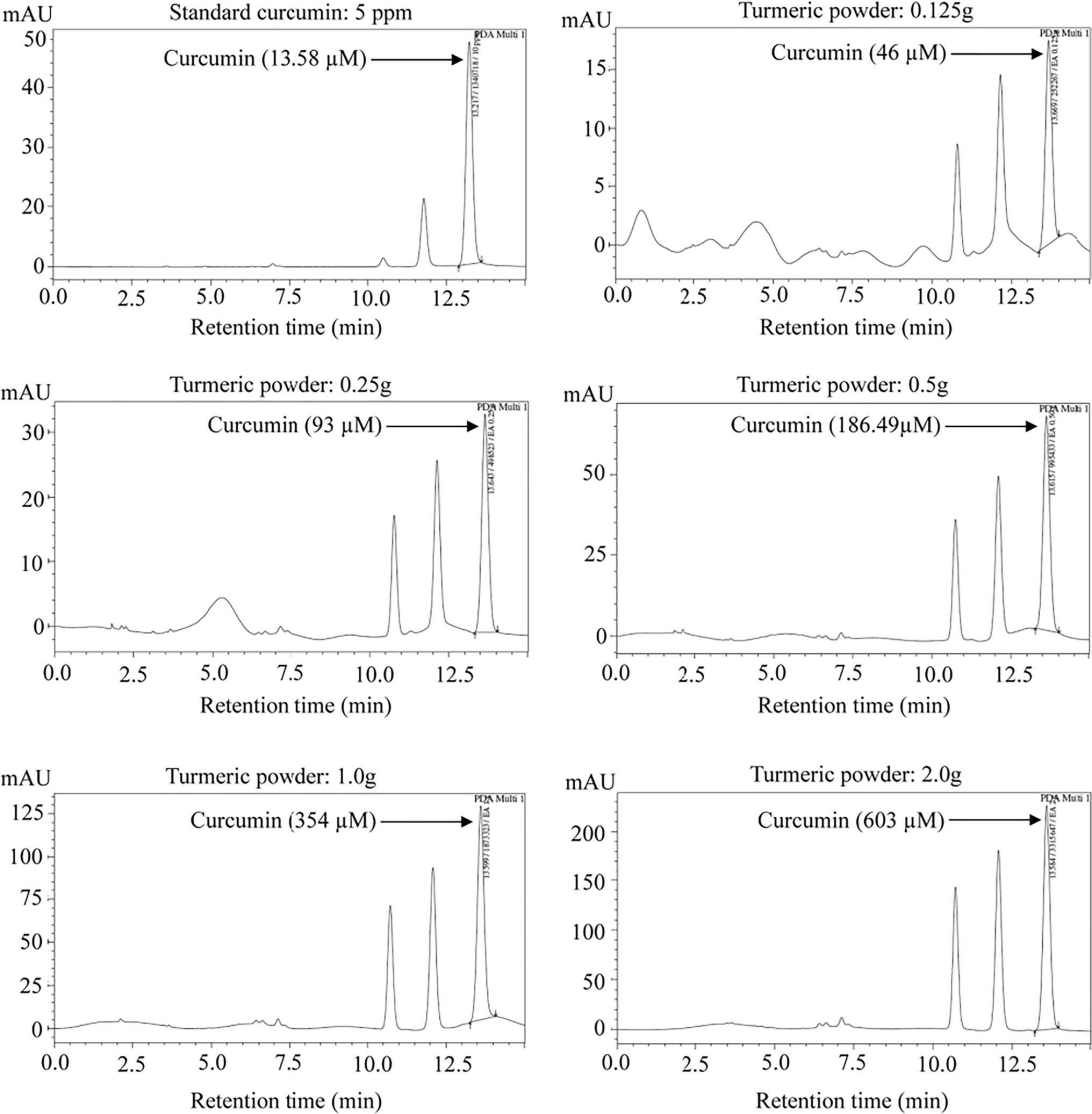
HPLC chromatograms of different concentrations of turmeric powder. **A** Standard curcumin standard curcumin for quantification of curcumin content in different concentrations of turmeric powder. **B** 0.125 g; **C** 0.25 g; **D** 0.5 g; **E** 1.0 g; **F** 2.0 g turmeric powder. The peak of curcumin was found at retention time13.58 min at 425 nm wavelength in all concentrations of turmeric powder and standard curcumin

Curcumin is the major component of turmeric, which affects gene expression through epigenetic mechanisms. It has been shown that the diverse effects of curcumin depend on its concentration [41]. Therefore, we quantified curcumin content in the various concentrations (0.125%, 0.25%, 0.5%, 1.0%, and 2.0%) of turmeric powder used in this study. HPLC chromatograms of all concentrations of turmeric powder and standard curcumin powder resulted in three peaks at a retention time of 10.75, 12.25, and 13.58 min, respectively (Fig. 9). In the HPLC chromatogram, the peak at a retention time of 13.58 min is corresponding to curcumin, and the other two peaks at retention time 10.75 and 12.25 min likely indicate bisdemethoxycurcumin and demethoxycurcumin, respectively [42]. From the linear relationship between peak areas, the curcumin content in 0.0125 g, 0.25 g, 0.5 g, 1.0 g, and 2.0 g turmeric powder was 46 μM, 93 μM, 186.49 μM, 354 μM, and 603 μM, respectively.

## Discussion

In this study, we evaluated the hormetic effect and elucidated the optimal dose of dietary turmeric. Additionally, the effects of dietary turmeric on the level of the housekeeping protein, β-tubulin in both genders of the *Drosophila* brain, and the correlation of β-tubulin level with lifespan, locomotion, fertility, oxidative stress tolerance, and eye health were studied. Lower concentrations (0.25% and 0.5%) of turmeric contributed to slow down the aging process, improve locomotion, fertility, oxidative stress tolerance, eye health, and β-tubulin levels in the brain of both genders of *D. melanogaster*. The higher concentration of turmeric (1% and 2%) showed degenerative changes in β-tubulin level as well as in various physiological traits. In line with our findings, male and female *Drosophila* treated with various doses of rutin (flavonoid plant pigment) demonstrated that rutin promoted longevity at lower concentrations but was detrimental at higher concentrations, implying that rutin’s longevity-promoting action is hormetic. At lower concentrations, this plant pigment also improved locomotion and exhibited enhanced survival upon exposure to oxidative stress, along with the elevation of transcript levels of *dFoxO*, *MnSod*, *Cat*, *dTsc1*, *dTsc2*, *Thor*, *dAtg1*, *dAtg5*, and *dAtg7* and reduced transcript level of *dTor* [43]. In addition, it has been reported in several investigations that the positive or negative effects of the diet depend on the concentration of its constituents [6, 44]. Excessive nutrition is linked with severe health conditions and an enhanced aging mechanism [1, 6]. Although it did not investigate hormetic effects, one particular study showed that anise hyssop (*Agastache foeniculum*) leaf powder extended the lifespan in a sex- and genotype-independent manner, increased fecundity, resistance to oxidative stress and starvation, enzymatic activities of superoxide dismutase, and aconitase, as well as elevated protein in *Drosophila* [45]. These results are in line with those obtained in our study. Upon examining the totality of our results, we determined that 0.5% turmeric is an optimal dose.

mTOR is a highly conserved nutrient-sensing pathway. It has been found that reducing mTOR activity through diet and nutrition results in a longer lifespan, improved neuromuscular effectiveness, and the preservation of a healthy heart in the elderly [7]. Organism morphology and health are determined by the interaction of multiple genes and environmental factors. Considering this, it is not surprising that turmeric could affect the housekeeping gene for tubulin protein homeostasis increasing its activity when administered at the optimal dietary dose. Since housekeeping genes are not involved in nutrient-sensing pathways, it is possible that turmeric affects post-translational modification and stabilization of tubulin through activity modulation of tubulin modifying enzymes. It has been well established that hyperphosphorylation of tau causes disassociation of tau from microtubules and tau aggregation, inducing tauopathy involved in neuronal dysfunction and brain aging. In the *C. elegans* study, it has been found that the relative amount of tubulin-unbound tau (free tau) was increased in high-expressing tau-transgenic *C. elegans*, leading to tau toxicity. The knockdown of a subset of tubulin led to enhanced tau toxicity even in low-expressing tau-transgenic *C. elegans*, and the suppression of tau expression in tubulin knockdown *C. elegans* rescued neuronal dysfunction [27]. These results indicate that reduction in tubulin can trigger the neurotoxicity of tau. Hence, the balanced level of tubulin and tau is indispensable to protect from tauopathy caused by abnormal tau depositions in the neuron. Moreover, losses of microtubules (MTs) and tubulin have been reported in the neurons of AD brains [46, 47], and this MT loss was found in neurons without neurofibrillary tangle formations, indicating that MT loss occurs independently of or ahead of the tangle formation. The experimental results showed that an optimal concentration of turmeric improved the β-tubulin protein level in the *Drosophila* brain (Fig. 4), which may counteract this imbalanced state and therefore elicit healthy aging. Our results of age-dependent changes in the levels of β-tubulin demonstrate that β-tubulin decreases with age in the brain *D. melanogaster*, indicating that β-tubulin is related to the aging process. Similarly, another study showed a decline in the level of β-tubulin in the primary human visual cortex during the progression of age [25], further supporting the correlation between tubulin and aging. Lifespan, locomotion and fertility, resistance to oxidative stress, and eye health decrease results showed an excellent correlation with tubulin decrease in the brain with age progression of *Drosophila*. It is unequivocal that the brain is the controller of the whole body. Tubulin protein is the building block of the microtubule filament, which is the key component of the cytoskeleton required for the shape and stabilization of neurons, outgrowth of axon and dendrites, muscular strength, and connection for cellular transportation and cell division [39]. Thus, maintaining constant tubulin level should contribute to proper brain structure and function increasing overall health. The optimal dose (0.5%) of turmeric improved the level of β-tubulin in the brain of young and middle-aged *D. melanogaster*. In addition, it has also been shown that this concentration of turmeric significantly inhibits the loss of β-tubulin protein after midlife in *Drosophila*. This indicates that turmeric could maintain the level of β-tubulin protein in the brain, thereby contributing to a more healthy aging process. Our observations are consistent with those suggesting that neuronal structural changes contribute to altered interneuronal connections as a result of exhausted tubulin resulting in deficiencies in brain function that have detrimental effects on the neuropathology of aging [26]. Evidence likewise exists suggesting that the loss of neuronal tubulin protein contributes to dimensional loss of neural processes bringing about changes such as brain aging and excessive alcoholism [26].

Since the brain uses a substantial amount of oxygen for its intense metabolic activities, it can generate free radicals and damages neuronal proteins [48, 49]. Therefore, the loss of neuronal tubulin can be induced by oxidative stress. Our understanding is that the antioxidant properties of turmeric maintain tubulin protein level by neutralizing oxidative stress. Consistent with this result, it has been reported that oxidative stress-induced proteomic damage has been avoided through the dietary antioxidants [50]. Visual problems and cortical blindness occur because of impairment in the visual cortex of the brain and a decrease in β-tubulin level in the visual cortex of the human brain during aging [25]. Since the reduction of tubulin level correlated with the damaging of the visual cortex, it would be reasonable to demonstrate that turmeric has a protective effect on the eye by maintaining the level of tubulin in the brain. Our results contribute to the advancement of developing an anti-aging agent for humans by targeting tubulin protein. Moreover, this work shows an interaction of dietary turmeric in vivo*.*

## Conclusion

Our experimental findings demonstrated that turmeric has a dual effect: at optimal concentrations, it exhibits beneficial effect, while at higher concentrations, it exhibits a detrimental effect. 0.5% turmeric is the optimal dose delaying the aging process through the maintenance of β-tubulin protein level in the *Drosophila* brain. Determining the optimal dose of turmeric is essential for the development of pharmaceutical agents aimed at targeting tubulin protein for increasing longevity as well as nutraceutical supplements for improving health. Our results also link turmeric to housekeeping and tau pathways by which dietary turmeric may modulate overall physiological traits for healthy aging. However, further research is warranted for elucidating the broad effects of turmeric on tubulin protein at the molecular level. Additionally, epidemiological studies and clinical trials are necessary to assess the attributes of turmeric’s dose-dependent effects in humans.

## Supplementary Information

Below is the link to the electronic supplementary material.

Supplementary file1 (DOCX 229 KB)

Supplementary file2 (DOCX 14 KB)

Supplementary file3 (XLSX 105 KB)

Supplementary file4 (DOCX 20 KB)

## Data Availability

Not applicable.

## Abbreviations

AD: Alzheimer’s disease
Aβ: Amyloid β
CAD: Carbamoyl-phosphate synthetase 2
DPPH: 2,2-Diphenyl-1-picrylhydrazyl
GAPDH: Glyceraldehyde 3-phosphate dehydrogenase
IIS: Insulin/IGF-1 signaling
miRNA: MicroRNA
MT: Microtubules
NO: Nitric oxide
ONOO¯: Peroxynitrite
PD: Parkinson’s disease
SH: Cysteine thiol groups
SOD: Superoxide dismutase
TOR: Target of rapamycin
