# Supplementary material for: Curcuma longa L. Prevents the Loss of β-Tubulin in the Brain and Maintains Healthy Aging in Drosophila melanogaster
Source: Mol Neurobiol. 2022 Jan 13;59(3):1819–35. doi: 10.1007/s12035-021-02701-6 (PMC8882102; doi:10.1007/s12035-021-02701-6)
Supplement: Supplementary file 1 — Supplementary file1 (DOCX 229 KB) [file 12035_2021_2701_MOESM1_ESM.docx]

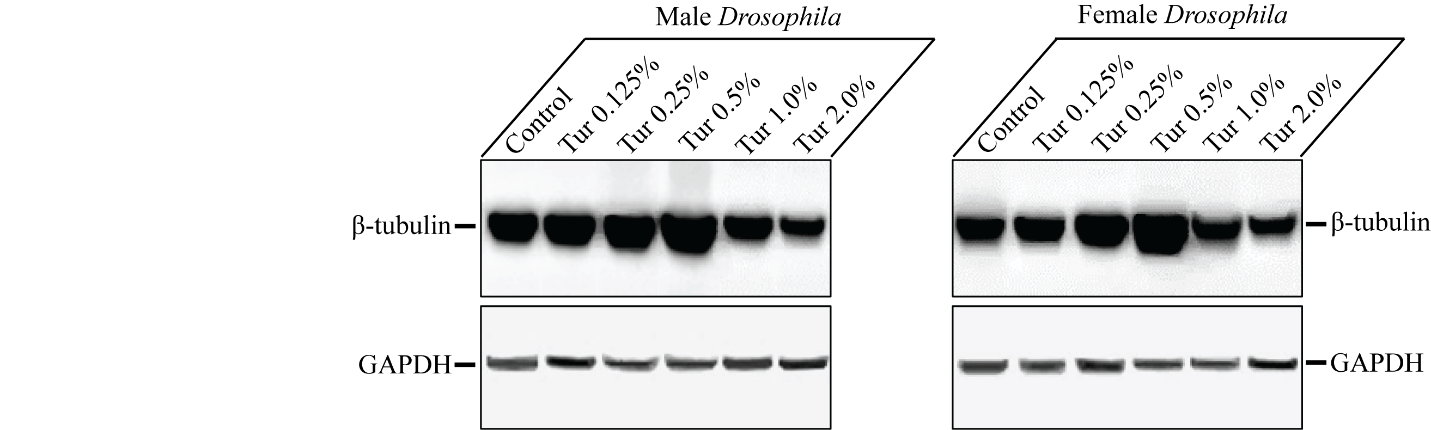


**Figure S1.** The biphasic effect of the doses of turmeric on β-tubulin protein level. (A) The β-tubulin level in both gender of *Drosophila* (upper panel) gradually increased as the turmeric concentration increased up to 0.5%, and the highest level of β-tubulin observed at 0.5% turmeric supplemented diet. The β-tubulin level was decreased gradually at the concentrations of 1% and 2% turmeric. The level of tubulin observed on the 30th day and determined by rabbit anti-β-tubulin primary antibody. GAPDH as loading control showed in the lower panel. There was no major discrepancy in GAPDH level in both gender of *Drosophila* reared on a regular diet, and 0.125%, 0.25%, 0.5%, 1.0%, and 2.0% turmeric supplemented diets.
