# Supplementary material for: Curcuma longa L. Prevents the Loss of β-Tubulin in the Brain and Maintains Healthy Aging in Drosophila melanogaster
Source: Mol Neurobiol. 2022 Jan 13;59(3):1819–35. doi: 10.1007/s12035-021-02701-6 (PMC8882102; doi:10.1007/s12035-021-02701-6)
Supplement: Supplementary file 2 — Supplementary file2 (DOCX 14 KB) [file 12035_2021_2701_MOESM2_ESM.docx]

**Table S1.** Media compositions used to maintain the *Drosophila melanogaster*.

| **Ingredient** | **Regular diet**  **(Control)** | **Turmeric powder supplemented diet** | | | | |
| --- | --- | --- | --- | --- | --- | --- |
|  |  | **Tur 0.125%** | **Tur 0.25%** | **Tur 0.5%** | **Tur 1.0%** | **Tur 2.0%** |
| Corn meal (g/L) | 84.00 | 84.0 | 84.0 | 84.00 | 84.0 | 84.0 |
| Active dry yeast (g/L) | 24 | 24 | 24 | 24 | 24 | 24 |
| Sucrose (g/L) | 47 | 47 | 47 | 47 | 47 | 47 |
| Agar (g/L) | 8 | 8 | 8 | 8 | 8 | 8 |
| 10% p-Hydroxy-benzoic  acid methyl ester (mL/L) | 5 | 5 | 5 | 5 | 5 | 5 |
| Propionic acid (mL/L) | 4 | 4 | 4 | 4 | 4 | 4 |
| Turmeric powder (g/L) | 0.0 | 1.25 | 2.5 | 5 | 10 | 20 |
