# Supplementary material for: Curcuma longa L. Prevents the Loss of β-Tubulin in the Brain and Maintains Healthy Aging in Drosophila melanogaster
Source: Mol Neurobiol. 2022 Jan 13;59(3):1819–35. doi: 10.1007/s12035-021-02701-6 (PMC8882102; doi:10.1007/s12035-021-02701-6)
Supplement: Supplementary file 4 — Supplementary file4 (DOCX 20 KB) [file 12035_2021_2701_MOESM4_ESM.docx]

**Table S3. Statistical analysis and comparison of median life span for male and female flies on control diet or diets supplemented with various concentrations of turmeric powder**

|  |  | Control | Tur 0.125 % | Tur 0.25 % | Tur 0.5 % | Tur 1 % | Tur 2 % |
| --- | --- | --- | --- | --- | --- | --- | --- |
| *w1118* (Male) | Median (95% C.I.) | 17.0 ~ 17.0 | 17.0 ~ 17.0 | 21.0 ~ 21.0 | 21.0 ~ 21.0 | 15.0 ~ 15.0 | - ~ - |
|  | Difference (%) |  | 0 | + 24 | + 24 | -12 |  |
|  | Log Rank test χ^2^ |  | 0.09 | 80.44 | 124.58 | 31.56 | 181.06 |
|  | Log Rank test p value |  | 0.7615 | 0 | 0 | 0 | 0 |
|  | Log Rank test Bonferroni p value |  | 1 | 0 | 0 | 0 | 0 |
|  | Fisher’s Exact test p value at 90% |  | 0.8154 | 2.20e-12 | 2.50e-12 | 1.40e-7 | 1.70e-12 |
| *w1118* (Female) | Median (95% C.I.) | 17.0 ~ 17.0 | 17.0 ~ 17.0 | 21.0 ~ 21.0 | 23.0 ~ 23.0 | 15.0 ~ 15.0 | - ~ - |
|  | Difference (%) |  | 0 | + 24 | + 35 | -12 |  |
|  | Log Rank Test χ^2^ |  | 0.39 | 46.89 | 109.54 | 32.8 | 238.15 |
|  | Log Rank test p value |  | 0.5321 | 0 | 0 | 1e-8 | 0 |
|  | Log Rank test Bonferroni p value |  | 1 | 0 | 0 | 5.1e-8 | 0 |
|  | Fisher’s Exact test p value at 90% |  | 0.6523 | 4.20E-07 | 2.10E-12 | 5.70E-09 | 2.10E-12 |

The survivability of cohorts was compared using a Log rank test for survival curves comparisons and Fisher's exact test with Bonferroni corrections to estimate differences in survival percentiles and medial lifespan. The experimental data were represented as the average ± SE of three replicates. Tur, Turmeric powder.
